# Rape in Nigeria: a silent epidemic among adolescents with implications for HIV infection

**DOI:** 10.3402/gha.v7.25583

**Published:** 2014-08-22

**Authors:** Morenike O. Folayan, Morolake Odetoyinbo, Abigail Harrison, Brandon Brown

**Affiliations:** 1 Institute of Public Health, Obafemi Awolowo University, Ile-Ife, Nigeria, Department of Child Dental Health, Obafemi Awolowo University, Ile-Ife, Nigeria; 2 Positive Action for Treatment Access, Lagos, Nigeria; 3 Department of Behavioral and Social Sciences, Brown University School of Public Health Providence, RI, USA; 4 Department of Population Health & Disease Prevention, University of California, Irvine, CA, USA

Adolescents worldwide often face tremendous sexual violence; a growing problem and a leading reproductive health concern. The prevalence of this violence ranges between 15 and 40% in sub-Saharan Africa, with studies showing rates of sexual coercion and abuse among female adolescents in Nigeria between 11 and 55% (1, 2). Little is known about the impact of rape on adolescents living with HIV (ALHIV), and how their HIV status affects how they cope with this traumatic experience. A recent survey in Nigeria showed 31.4 and 5.7% of sexually active adolescent females and males, respectively, reported forced sex (rape) at sexual initiation (3). The same study showed significantly more reported cases of rape among female ALHIV compared to their HIV-negative peers (*p*=0.008). Supporting literature from South Africa highlights rape as a risk factor for HIV in women (4). Achunike and Kitause provide vivid accounts of rape in Nigeria and its impact on victims, including physical injuries, fatigue and chronic headaches, and emotional problems, such as suicide attempts, stress disorders, depression, and sexual dysfunction (5, 6). Alcohol and drug abuse were also prominent for victims. In addition, adolescents and youths who have been sexually abused are more likely to have multiple concurrent sex partners, are less likely to report using contraception, and are more likely to report pregnancy (6).

Prior reports have shown that 4–6% of all adolescent girls in southwestern Nigeria experience rape (3, 7). The strict code of silence among victims implies the potential for under-reporting, especially when victim blaming is the norm. Less than one in five (18.1%) of 10,000 respondents who have been raped in Nigeria report the offence to the police (8). There are many reasons for this. Rape results in stigmatization of the victim, resulting in rejection by families and communities, and with police sometimes unwilling to make official reports. Due to this stigma, women and adolescents may be unwilling or unable to obtain a medical examination to substantiate their report of rape (8). High rates of rape and low reporting underscore the need for specific actions to address sexual violence and to stem the tide on potential risks of HIV transmission. A report by Sinclair et al. showed that building self-defense skills of girls in Kenya significantly reduced incidence of rape over a 10-month period (9). However, prior to planning and implementing a similar program in Nigeria, it is important that leadership recognizes there is a rape epidemic. HIV infection is just one of the multiple challenges rape victims face, but a strong reason for stakeholders engaged with HIV prevention programmes to incorporate rape prevention in current and proposed HIV prevention programmes for adolescents.

Olatunji conducted an extensive review of the Nigerian anti-rape law and identified shortcomings in the provisions which make rape prevention challenging (10). First, according to the law, rape can only be committed by a man to a woman, and it involves only penal and vaginal sex. The law does not acknowledge male rape victims nor does it recognize anal sex as part of rape. Second, a victim of rape needs to establish that penetration occurred, corroboration (or validation) of the crime needs to be established, and proof must be provided that consent was not given. The limitations with establishing consent make proving many of the few valid rape cases difficult (10). Overall, the low prospect of receiving legal judgment for rape stifles enthusiasm in seeking legal recourse.

The recent rape and abduction of 276 female adolescents in Nigeria have further stirred up discussions and media attention about rape of girls and women within the context of conflict in the country (Table 1). Unfortunately, there is still little public dialogue linking rape and HIV infection, even when rape is occurring among married couples. The general population, government, and lawmakers need to understand the epidemic proportions of the crime and its potential long-term impact on the health of victims. This will help facilitate more structured interventions for the prevention of HIV among female adolescents in Nigeria.

**Table 1:** Short list of reported rape in Nigerian media

Source	Month–year	Article topic
Independent Television & Radio	July 2013	Increase in rape cases in Nigeria
Gist Ville	October 2013	18-year old girl commits suicide after gang rape in Bayelsa
Punch	February 2014	Tackling the rape epidemic in Nigeria
HmmNaija.com	February 2014	12-year-old rape victim: I'm glad he had an accident after raping me
Vanguard	March 2014	Obesere rape saga-case transferred to SCID
Nigerian Tribune	April 2014	Over 80 rape cases recorded in Edo State in 7 months
Premium times	April 2014	Checking high incidence of rape in Nigeria
Channels TV	April 2014	Ondo Police Record 45 Rape Cases in 2013
Scan News	April 2014	ICC moves against rape in Nigeria
The Paradigm	May 2014	Two Chibok girls raped and left to die In Sambisa Forest By Boko Haram
DailyPost	May 2014	Gunmen invade Benue University, rape 20 female students
News 24 Nigeria	June 2014	UN: Nigerian schoolgirls face rape danger
Naija Standard Newspaper	July 2014	Policeman 32, rapes JSS 1 virgin girl

*Morenike O. Folayan* Institute of Public Health Obafemi Awolowo University, Ile-Ife, Nigeria Department of Child Dental Health Obafemi Awolowo University, Ile-Ife, Nigeria *Morolake Odetoyinbo* Positive Action for Treatment Access, Lagos, Nigeria*Abigail Harrison* Department of Behavioral and Social Sciences Brown University School of Public Health Providence, RI, USA *Brandon Brown* Department of Population Health & Disease Prevention University of California, Irvine, CA, USA Email: brandon.brown@uci.edu
